# Usability of rectal swabs for microbiome sampling in a cohort study of hematological and oncological patients

**DOI:** 10.1371/journal.pone.0215428

**Published:** 2019-04-15

**Authors:** Lena M. Biehl, Debora Garzetti, Fedja Farowski, Diana Ring, Martin B. Koeppel, Holger Rohde, Philippe Schafhausen, Bärbel Stecher, Maria J. G. T. Vehreschild

**Affiliations:** 1 University of Cologne, Department I of Internal Medicine, University Hospital of Cologne, Cologne, Germany; 2 Institute for Medical Microbiology, Virology and Hygiene, University Medical Center Hamburg-Eppendorf, Hamburg, Germany; 3 German Centre for Infection Research (DZIF), Partner site Bonn-Cologne, Cologne, Germany; 4 Max von Pettenkofer Institute, Faculty of Medicine, LMU Munich, Munich, Germany; 5 German Centre for Infection Research (DZIF), Partner site Munich, Munich, Germany; 6 German Centre for Infection Research (DZIF), Partner site Hamburg-Borstel, Hamburg, Germany; 7 Department of Oncology and Hematology, Hubertus Wald Tumorzentrum/University Cancer Center Hamburg, University Medical Center Hamburg-Eppendorf, Hamburg, Germany; 8 Infectious Diseases Unit, Medical Clinic II, University Hospital Frankfurt, Frankfurt am Main, Germany; Medical University of South Carolina, UNITED STATES

## Abstract

**Objectives:**

Large-scale clinical studies investigating associations between intestinal microbiota signatures and human diseases usually rely on stool samples. However, the timing of repeated stool sample collection cannot be predefined in longitudinal settings. Rectal swabs, being straightforward to obtain, have the potential to overcome this drawback. Therefore, we assessed the usability of rectal swabs for microbiome sampling in a cohort of hematological and oncological patients.

**Study design:**

We used a pipeline for intestinal microbiota analysis from deep rectal swabs which was established and validated with test samples and negative controls. Consecutively, a cohort of patients from hematology and oncology wards was established and weekly deep rectal swabs taken during their admissions and re-admissions.

**Results:**

Validation of our newly developed pipeline for intestinal microbiota analysis from rectal swabs revealed consistent and reproducible results. Over a period of nine months, 418 rectal swabs were collected longitudinally from 41 patients. Adherence to the intended sampling protocol was 97%. After DNA extraction, sequencing, read pre-processing and filtering of chimeric sequences, 405 of 418 samples (96.9%) were eligible for further analyses. Follow-up samples and those taken under current antibiotic exposure showed a significant decrease in alpha diversity as compared to baseline samples. Microbial domination occurred most frequently by Enterococcaceae (99 samples, 24.4%) on family level and *Enterococcus* (90 samples, 22.2%) on genus level. Furthermore, we noticed a high abundance of potential skin commensals in 99 samples (24.4%).

**Summary:**

Deep rectal swabs were shown to be reliable for microbiome sampling and analysis, with practical advantages related to high sampling adherence, easy timing, transport and storage. The relatively high abundance of putative skin commensals in this patient cohort may be of potential interest and should be further investigated. Generally, previous findings on alpha diversity dynamics obtained from stool samples were confirmed.

## Introduction

The intestinal tract harbors a complex microbial community which plays a central role in human health. Disruption of the gut microbiota (or dysbiosis) is associated with pathological intestinal conditions such as obesity and malnutrition, metabolic diseases such as diabetes, and chronic inflammatory diseases such as inflammatory bowel diseases [1–4]. The recent increase in clinical microbiome studies has been facilitated by advances in high throughput sequencing technologies, which offer rapid and comprehensive culture-independent techniques, mostly relying on DNA sequencing of the 16S ribosomal RNA gene [5].

When conducting clinical studies addressing microbiome dynamics in humans, longitudinal sampling at precisely determined intervals is often needed. Except for settings where mucosal biopsies are available, such as during colonoscopies, stool samples are the “gold standard” starting material for this purpose [6]. However, their acquisition can be challenging. Most importantly, timing can be jeopardized if a study participant does not produce a stool sample within the desired time window. Furthermore, immediate transport and adequate storage is difficult during evenings and on weekend days. These organizational drawbacks may be overcome if rectal swabs are used instead. In fact, they can be obtained at specific time points, thus preventing prolonged periods between sampling and storage. As shown by previous studies, microbiota profiles of rectal swab samples are, to some extent, similar to the ones of fecal samples, even though contamination with skin bacteria can occur and may be caused by incorrect sample collection [6–9]. To our knowledge, so far only few studies have used rectal swabs for microbiota sampling in a clinical setting. Published work mainly focused on healthy subjects and on patients with inflammatory bowel disease [6, 10]. Therefore, little is known about the applicability of rectal swabs during chemotherapy and antibiotic treatment, interventions which are both known to affect the intestinal microbiome [11, 12]. In this study we report on the usability of rectal swabs for microbiota sampling in a longitudinal cohort of high-risk hematological and oncological patients undergoing chemotherapy and antibiotic treatment.

## Materials and methods

### Validation and control samples

In order to validate our pipeline for microbiome analysis and its application on rectal swabs, we tested its performance and reproducibility on a small number of subjects (S1 Table). Stool and deep rectal swab samples (FecalSwab^TM^, COPAN Italia, Brescia, Italy) were obtained from one patient and seven healthy volunteers. For each individual, specimens were collected simultaneously. To account for different sampling times, storage conditions, primer pairs and DNA extraction batches, we included technical replicates for volunteer H1 (S1 Table). Moreover, as the transport medium may impact microbiome profiling at several steps and influence bacterial survival/growth, DNA extraction and PCR efficiency, we obtained a second deep rectal swab from four of the volunteers immediately after the first swabWhile a Cary-Blair medium (composition: Na_2_HPO_4_ 1.1 g/l, C_2_H_3_NaO_2_S 1.5 g/l, NaCl 5.0g/l, CaCl_2_ 0.09 g/l, agar 5.6 g/l) is used as a standard solution in our swabs, the second swabs were transported and frozen in saline solution (0.9% NaCl). In order to exclude errors which may arise during any step of our microbiome analysis pipeline, we included a mock community composed of twelve bacterial strains [13]. In detail, 200 μl of the mock community were inoculated either in 1 ml Cary Blair medium or 1 ml saline solution, and processed in the same way as the other validation samples.

PCR, sequencing and Bioinformatic analyses were performed as described in the paragraph “Bioinformatic analyses and taxonomic profiling”, except that a rarefaction level of 10,000 sequences per sample was applied before alpha and beta diversity calculation. Correlation analysis of taxonomy profiles was done calculating R^2^ correlation coefficients for all pairs of samples, while statistics significance was calculated with a 2-way ANOVA test with a Bonferroni post hoc test.

To monitor possible contaminations, the pipeline was also applied to experimental controls from sterile swabs, DNA extraction buffers and reagents used for amplicon library preparation, which were processed together with the patient samples. For these negative control samples, sequencing was performed with specific primers (see below and S2 Table) and taxonomic classification was performed aligning the pre-processed high-quality and chimera-free sequences to the SILVA databases with the online tool SILVA Incremental Aligner, version 1.2.11 [14].

### Patients and study settings

Patients were eligible for inclusion into the longitudinal cohort if they were admitted for intensive cytotoxic treatment with subsequent neutropenia requiring an expected hospitalization of at least 3 weeks. The cohort study was approved by the local institutional review board and ethics committee of the Medical Chamber in Hamburg (Ethik-Kommission der Ärztekammer Hamburg), Germany (Study ID: PV4722). Samples from healthy volunteers were collected within a volunteer sample collection protocol approved by the Ethics Commission of the Faculty of Medicine of Cologne University (Ethikkommission der Medizinischen Fakultät der Universität zu Köln), Germany (Study ID: 16–234). Written informed consent was obtained from all patients and healthy volunteers prior to inclusion into the study.

All patients were treated according to local standards of care at the Medical Clinic and Polyclinic of the University Medical Center Hamburg-Eppendorf, Germany, which includes antimicrobial prophylaxis with low-dose trimethoprim/sulfamethoxazole twice weekly and ciprofloxacin or sultamicillin daily during neutropenia.

### Clinical data and definitions

Clinical data collected from all patients included underlying disease, chemotherapy, antibiotic prophylaxis and treatment, duration of neutropenia (defined as a neutrophil count below 500/μl), abdominal symptoms, temperature, as well as results of microbiological tests. Samples under current antibiotic exposure were defined as samples taken during ongoing antibiotic administration of at least 3 days prior sampling or samples taken within 2 days after termination of antibiotic treatment.

### Sample collection and procession

Deep rectal swabs (FecalSwab, COPAN Italia, Brescia, Italy, lot number N02T01) were obtained at baseline and weekly during inpatient treatment. Sampling was resumed in case of re-admission until no further oncological treatment was planned or until the maximum study duration of 9 months. Deep rectal swabs were performed by trained medical staff (nurses, medical students), with swabs being inserted at least 2 cm intrarectally and rotated while avoiding contact with the perianal skin of the patient.

After sample collection, storage and transport of the fecal swabs were allowed at room temperature for a maximum of 4 hours or at 4–8°C for a maximum of 5 hours. Swab Cary-Blair media (2,000 μl) were vortexed, and aliquots of 1,200 μl were stored at -80°C until microbiome analysis, while the remaining 800 μl were kept at -80°C for any further necessary experiment.

### Microbiota analysis

Profiling of the gut microbiota was performed as recently published [15] with some modifications, as described below.

#### DNA isolation

Genomic DNA (gDNA) was isolated from rectal swabs according to the procedures previously described [16, 17]. Briefly, each frozen sample was thawed, pelleted and suspended in 500 μl of extraction buffer (200 mM Tris pH 8.0, 200 mM NaCl, 20 mM EDTA), 210 μl of 20% SDS, 500 μl of a mix of phenol:chloroform:isoamyl alcohol (24:24:1), and 500 μl of zirconia/silica beads (0.1-mm diameter). Bacterial cells were lysed by mechanical disruption with a bead beater (50 s^-1^) for 4 min, followed by DNA extraction in phenol:chloroform:isoamyl alcohol and precipitation with ethanol. The isolated gDNA was resuspended in 10 mM Tris buffer and purified with NucleoSpin gDNA clean-up columns (Macherey-Nagel, Düren, Germany) in a final elution volume of 50 μl. gDNA was finally quantified by a fluorometric method (Quant-iT PicoGreen dsDNA Reagent, Thermo Fisher Scientific, Darmstadt, Germany), and stored at 4°C before further processing.

#### 16S rRNA amplicon sequencing

For microbiota profiling, a dual-index strategy multiplexing 8 forward and 12 reverse primers for 16S rRNA gene sequencing on the MiSeq Illumina platform was developed, as previously described [18]. PCR amplification primers targeting the V3-V4 variable regions of the 16S rRNA gene were designed by Eurofins Genomics (Ebersberg, Germany) using a proprietary layout based on established methods (S2 Table) [19, 20]. To monitor possible errors due to contaminations or read-to-sample miss-assignment, primers for 8 control samples with specific barcodes were also included (S2 Table). PCR reactions contained 1X Q5 Hot Start High-Fidelity Master Mix (New England BioLabs, GmbH, Frankfurt Am Main, Germany), 0.5 μM each forward and reverse primers, and 15–20 ng of genomic DNA template. Reactions consisted of 30 s at 94°C; 30 cycles of 10 sec at 95°C, 20 sec at 55°C and 10 sec at 72°C; and a final extension at 72°C for 2 min. Each sample was amplified in duplicate and purified using the Agencourt AMPure XP PCR Purification system (Beckman Coulter, Krefeld, Germany). Purified amplicons were quantified using the fluorometric PicoGreen dsDNA reagent. A pooled sample was created by combining equimolar amounts of the 96 individual amplicons and sent to Eurofins Genomics for library quality control and sequencing. Libraries were mixed with 1% PhiX genomic control library and sequenced on the Illumina MiSeq v3 as 300-bp paired-end runs.

#### Bioinformatic analyses and taxonomic profiling

Prior to taxonomic analysis, reads were pre-processed to retain only high-quality sequences, using a combination of scripts from the BBTools package [http://jgi.doe.gov/data-and-tools/bbtools/] and Trimmomatic [21] (see S1 File for a full list of used commands). Reads had to possess the full barcode free of errors, be longer than 200 bp after removal of the variability region and quality trimming, and have a minimum of 30 bp overlap with no mismatches during merging of paired-end reads. Joined reads, which had to be longer than 400 bp, were then quality filtered during demultiplexing with QIIME v. 1.9 [22], allowing a minimum Phred quality score of 20 and no ambiguous bases. Failed sequencing was considered for samples having a number of reads lower than the negative sample(s) included in the run. Chimeric sequences were then removed with USEARCH v. 6.1 [23]. Samples containing less than 2,000 sequences were heuristically considered not adequate for accurate profiling of the bacterial community composition and omitted for subsequent analyses. OTU (Operational Taxonomic Unit) picking and taxonomic classification were performed with QIIME. Open-reference OTU clustering and taxonomy assignment of sequences were performed with UCLUST [23] against the Silva database Release 119 [24] at the 97% similarity level. OTUs with a number of sequences < 0.01% of the total number of sequences were discarded as a second level of quality-filtering. After conducting a rarefaction test to determine the sufficient number of sequences for an accurate estimation of species diversity, the OTU table was finalized using a rarefaction level of 2,000 sequences per sample.

#### Statistical analysis

All further analyses were carried out using R for Statistical Computing version 3.2.5 (R Foundation for Statistical Computing, Vienna, Austria) [25]. The alpha and beta diversity metrics were calculated using functions provided in the phyloseq R package [26]. Continuous data was presented as mean (± standard deviation) and/or median (range) and tested with appropriate statistical analyses (two-tailed t-tests). Absolute and relative frequencies were given for categorical data (i.e. dominations) and tested using the chi-square test. Domination was defined as a single bacterial taxon comprising at least 30% of sequences and being the most abundant taxon [27].

### Presence of skin commensals

In order to evaluate whether detected bacterial taxa were originating from the anal skin rather than the rectal mucosa as a possible contamination introduced by inadequate sampling we investigated the presence of skin commensals [28]. From all detected taxa, the genera *Corynebacterium*, *Staphylococcus* and *Streptococcus* were chosen as potential contaminants and a relative abundance of more than 10% was defined as increased. Furthermore, frequencies of domination by these genera and a shift towards the dominating genus in the previous and subsequent samples were assessed. A shift was defined as an increased abundance of the respective genus of more than 10% in the sample directly prior or after the sample showing the domination. Of note, both terms, domination and shift only refer to results from compositional data and not to the total amount of bacteria in a sample.

### Availability of data and material

The datasets generated and analyzed during the current study are available in the NCBI Sequence Read Archive under the BioProject accession number PRJNA376506 (validation and patient samples). Sequences from the negative controls are available as fasta S2 File.

## Results

### Pipeline validation

We used a set of 35 samples for validation of our microbiota analysis pipeline and for evaluating reproducibility of the results. In particular, we designed experiments to control for effects of sample collection, transport media, gDNA extraction and 16S rRNA amplicon sequencing. The analysis included samples from a patient and seven healthy volunteers as well as a mock bacterial community with known composition (S1 Table). Overall, highly consistent and reproducible results were obtained, proving that our new pipeline allows microbiota analysis of complex human intestinal samples. Specifically, we obtained a high number of reads from both stools and rectal swabs (range 12,490–415,602, median 93,112). Although the gDNA concentration from stools was higher than that from rectal swabs, this difference was not maintained during PCR amplification nor reflected in the sequencing output (S1 Table).

**Fig 1:**
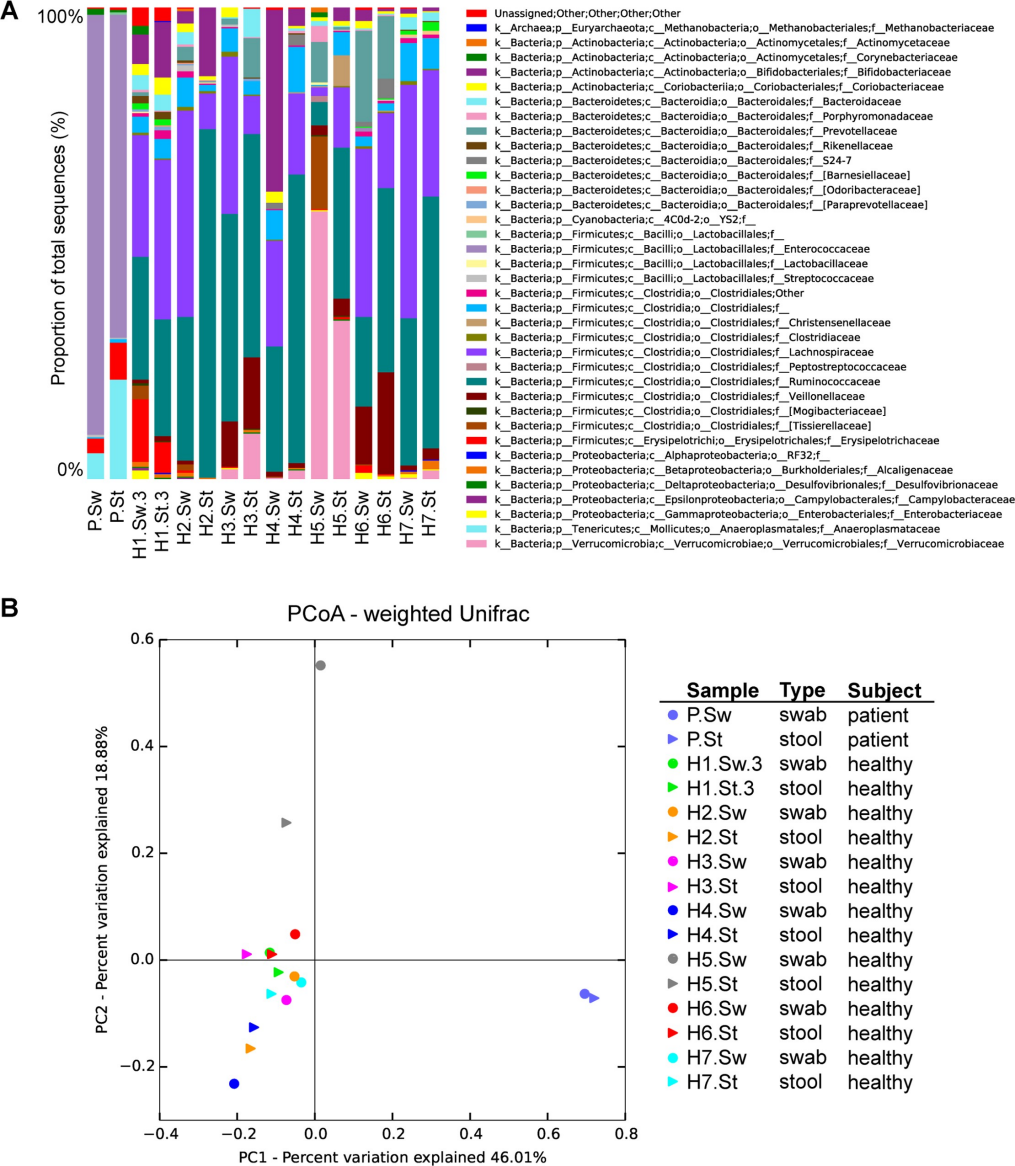
Comparison of stool- versus swab-derived microbiota profiles. **(**A) Taxonomic profiles at the family level. The legend has the same color direction as in the bar plots. (B) Beta-diversity calculated as weighted Unifrac and visualized by principal coordinate analysis (PCoA). P–patient; H–healthy volunteer; Sw–swab; St–stool.

Rectal swabs and stools sampled at the same time point from the same subject yielded similar results in terms of microbiota composition. This applied both, for the patient (R^2^ = 0.97, Shannon index: swab = 1.23, stool = 2.28) and for the healthy volunteers (R^2^ = 0.86, Shannon index mean: swabs = 5.53, stools = 5.29) (Fig 1). Besides overall similarities of the taxonomic profiles, few differences in the individual identified taxa were detected (Fig 1A). In particular, the relative abundance of Enterococcaceae was higher in the patient swab sample (*p*<0.01), and Anaeroplasmataceae were more abundant in the patient stool (*p*<0.05), while stool samples from the healthy volunteers showed a significantly lower abundance of Lachnospiraceae and a higher proportion of Ruminococcaceae, compared to the swab samples (*p*<0.001). As expected, the patient showed a completely different microbiota profile compared to the healthy subjects (Fig 1B). Interestingly, our validation samples did not reveal higher abundances of skin commensals in swabs compared to stools, as seen in the patient samples (see below). In fact, Corynebacteriaceae were only detected in the patient samples and in two healthy subjects (H1 and H5), while the Streptococcaceae family was present in very low abundance in all rectal swabs from healthy individuals (mean of all samples: 0.2%).

The reproducibility and consistency of the results were confirmed by analyzing microbiota profiles from technical replicates of the swabs and stools taken from volunteer H1 (S1 Table). Indeed, major variations due to extraction of the gDNA in three batches (R^2^ = 0.98), or the use of alternative primer pairs (R^2^ = 1), or different storage conditions of stools (R^2^ = 0.99), or sampling of swabs at two time points (R^2^ = 0.89) could be excluded (S1 Fig).

Likewise, any impact of the Cary-Blair medium on microbiome profiles was ruled out by comparison with saline solution as a transport medium in swabs from four volunteers and a mock bacterial suspension (S1 Table and S2 Fig). Importantly, both mock samples revealed the expected bacterial composition, with 12 bacterial genera and 10 families detected [13].

### Control samples

To control for possible sources of contaminating bacterial DNA, we included experimental negative controls from DNA extraction buffers, PCR reagents, and sterile swabs. Overall, 14 control samples were sequenced, generating between 0 and 298 reads per sample. After pre-processing and chimera filtering, a total of 860 sequences remained (S3 Table and S2 File). All sequences could be classified as belonging to the Bacteria kingdom, and for 492 (57.2%) and 66 (7.7%) sequences classification was possible at the genus and species level, respectively. The predominant contaminants detected in our control samples are summarized in Table 1. Mostly, they have been already identified as contaminants of DNA extraction kits, PCR reagents, laboratory consumables, water and personnel [29–32]. Of the still unreported contaminants, some are common environmental organisms (e.g. *Sediminibacterium*, *Legionellales*, *Cyanobacteria*, *Hydrogenophilus*), while others are skin/mucosa-associated bacteria (e.g. *Staphylococcus*, *Dermabacteraceae*). Sequences assigned to intestinal bacteria (e.g. *Enterococcus*, *Roseburia*, *Ruminococcus*) may have been introduced during sample processing. However, due to the very low number of sequences, they are unlikely to affect the analysis of patient microbiome profiles.

**Table 1:** List of the main contaminants detected in negative control samples.

Negative control	Phylum	Family	Genus	Proportion (%)^b^
**Sterile swabs**	Bacteroidetes	Flavobacteriaceae	*Flavobacterium*^a^	17.9
	Firmicutes	Enterococcaceae	*Enterococcus*	9.2
		Lachnospiraceae	Unclassified	5.8
	Proteobacteria	Rhizobiaceae	*Agrobacterium*^a^	5.8
		Pseudomonadaceae	*Pseudomonas*^a^	4.8
**DNA extraction buffers**	Actinobacteria	Corynebacteriaceae	*Corynebacterium*^a^	5.8
		Mycobacteriaceae	*Mycobacterium*^a^	4.6
	Armatimonadetes	[Fimbriimonadaceae]	Unclassified	5.8
	Proteobacteria	Comamonadaceae	*Schlegelella*^a^	5.2
			Unclassified^a^	5.8
		Enterobacteriaceae	Unclassified	6.4
		Legionellales, Unclassified	Unclassified	4.6
		Pseudomonadaceae	*Pseudomonas*^a^	4.6
**PCR reagents**	Armatimonadetes	[Fimbriimonadaceae]	Unclassified	5.2
	Bacteroidetes	Chitinophagaceae	*Sediminibacterium*	4.8
	Proteobacteria	Caulobacteraceae	Unclassified	5.6
		Brucellaceae	*Ochrobactrum*^a^	5.4
		Methylobacteriaceae	*Methylobacterium*^a^	9.2
		Comamonadaceae	Unclassified^a^	23.9
		Legionellales, Unclassified	Unclassified	6.3
		Pseudomonadaceae	*Pseudomonas*^a^	5.4

^a^Previously reported as contaminants [29–32]

^b^Proportion of sequences relative to each negative control type; only contaminants with proportions>4.5% are reported. For full list of contaminants, see S3 Table.

### Patient characteristics and sample collection

From July 2014 to March 2015, 41 patients were included into the study. Median age was 60 years (range 19–76) with 22 patients (53.7%) being female. Acute leukemia was the most frequent underlying disease (29 patients; 70.7%) followed by lymphoma (7; 17.1%) and solid tumor (3; 7.3%). One patient had suspected rectal carcinoma, which was not confirmed, and one patient was admitted for hematopoietic stem cell transplantation for treatment of Multiple Sclerosis. The median duration of longitudinal sampling was 55 days (range 1–246). All except one patient received antibiotic treatment during their study participation, while all except three patients received chemotherapy.

According to the sample collection protocol, 431 weekly rectal swabs were planned to be collected during all admissions and re-admissions of study patients. We were able to collect 418 samples (range 1–30 per patient), resulting in a completeness rate of 97.0%. Reasons for missed weekly samples were short inpatient stay of less than 3 days and rejection of sampling by the patient, in one case due to general weakness and tiredness, and in one case due to an anal fissure and anticipated pain during sampling.

No adverse events occurred during rectal swab sampling, especially no bleeding, despite the low platelet counts of as low as 10x10^9^/l and no consecutive infections, despite patients being neutropenic during some of the sampling. Two patients decided to quit their study participation due to their wish to have fewer interventions and fewer disturbances during their oncological treatment.

### DNA extraction from rectal swabs

Isolation of genomic DNA, as assessed by fluorometric quantification, generated an average DNA concentration of 7.4 ng/μl (range: -0.228–110 ng/μl) (S4 Table). Importantly, no relation was found between the genomic DNA amount and the concentration of the corresponding PCR amplicon or the read output.

### Microbiome analysis from rectal swabs

PCR amplicons obtained from all the 418 rectal swabs were achieved in five different runs (96 samples each, including negative controls). After read pre-processing and filtering of chimeric sequences, on average 85,106±60,725 sequences per sample were obtained (S4 Table). Samples that failed sequencing (n = 4), contained less than 2,000 reads after raw read pre-processing (6), or had less than 2,000 sequences represented in the OTU table used for calculation of diversity metrics (3) were omitted (total 13 out of 418 samples, 3.1%), resulting in a final collection of 405 samples with on average 87,811±59,752 sequences per sample (Table 2). Importantly, we observed a significantly higher amount of sequences (93,897±60,642 vs 67,950±52,297; p<0.001) in samples from patients without current antibiotic exposure (n = 310/405) (Table 3).

**Table 2:** Sequences per sample, alpha diversity scores and frequency of dominations.

	Total (n = 405)	Comparison		
		Baseline samples (day = 0; n = 39)	Post baseline samples (day>0; n = 366)	*p*-value
**Sequences per sample mean** ± SD	**87811** ± 59752	**57513** ± 37036	**91039** ± 60831	**<0.001**
(range)	(2787-279263)	(16620-170624)	(2787-279263)	
**Shannon index mean** ± SD	**2.28** ± 1.11	**2.68** ± 1.01	**2.24** ± 1.11	**0.013**
(range)	(0.01-4.46)	(0.10-4.23)	(0.01-4.46)	
**Inverse Simpson mean** ± SD	**8.02** ± 8.08	**9.55** ± 7.30	**7.86** ± 8.14	**0.180**
(range)	(1.00-48.74)	(1.04-26.52)	(1.00-48.74)	
**PD whole tree mean** ± SD	**12.21** ± 6.70	**16.72** ± 7.63	**11.73** ± 6.42	**<0.001**
(range)	(1.07-34.25)	(1.07-32.99)	(1.11-34.25)	
**Domination**				
any domination by family % (n)	**83.5** (338)	**71.8** (28)	**84.7** (310)	**0.066**
any domination by genus % (n)	**57.0** (231)	**48.7** (19)	**57.9** (212)	**0.350**
domination by *Enterococcus* % (n)	**22.2** (90)	**5.1** (2)	**24.0** (88)	**0.012**

SD: standard deviation; PD: phylogenetic diversity

**Table 3:** Effect of antibiotic exposure on sequences per sample and alpha diversity metrics.

	No current antibiotic exposure^a^ (n = 310)	Current antibiotic exposure^a^ (n = 95)	*p*-value
Sequences per sample mean ± SD	93897 ± 60642	67950 ± 52297	<0.001
Shannon index mean ± SD	2.50 ± 1.11	1.59 ± 0.76	<0.001
Inverse Simpson mean ± SD	9.43 ± 8.66	3.43 ± 2.57	<0.001
PD whole tree mean ± SD	13.34 ± 6.95	8.53 ± 4.03	<0.001

SD: standard deviation; PD: phylogenetic diversity

^a^Samples under current antibiotic exposure were defined as i) samples taken during an ongoing antibiotic exposure that started at least 3 days prior to sampling or ii) samples taken within 2 days after termination of antibiotic treatment.

### Bacterial diversity analysis of rectal swab samples

**Fig 2:**
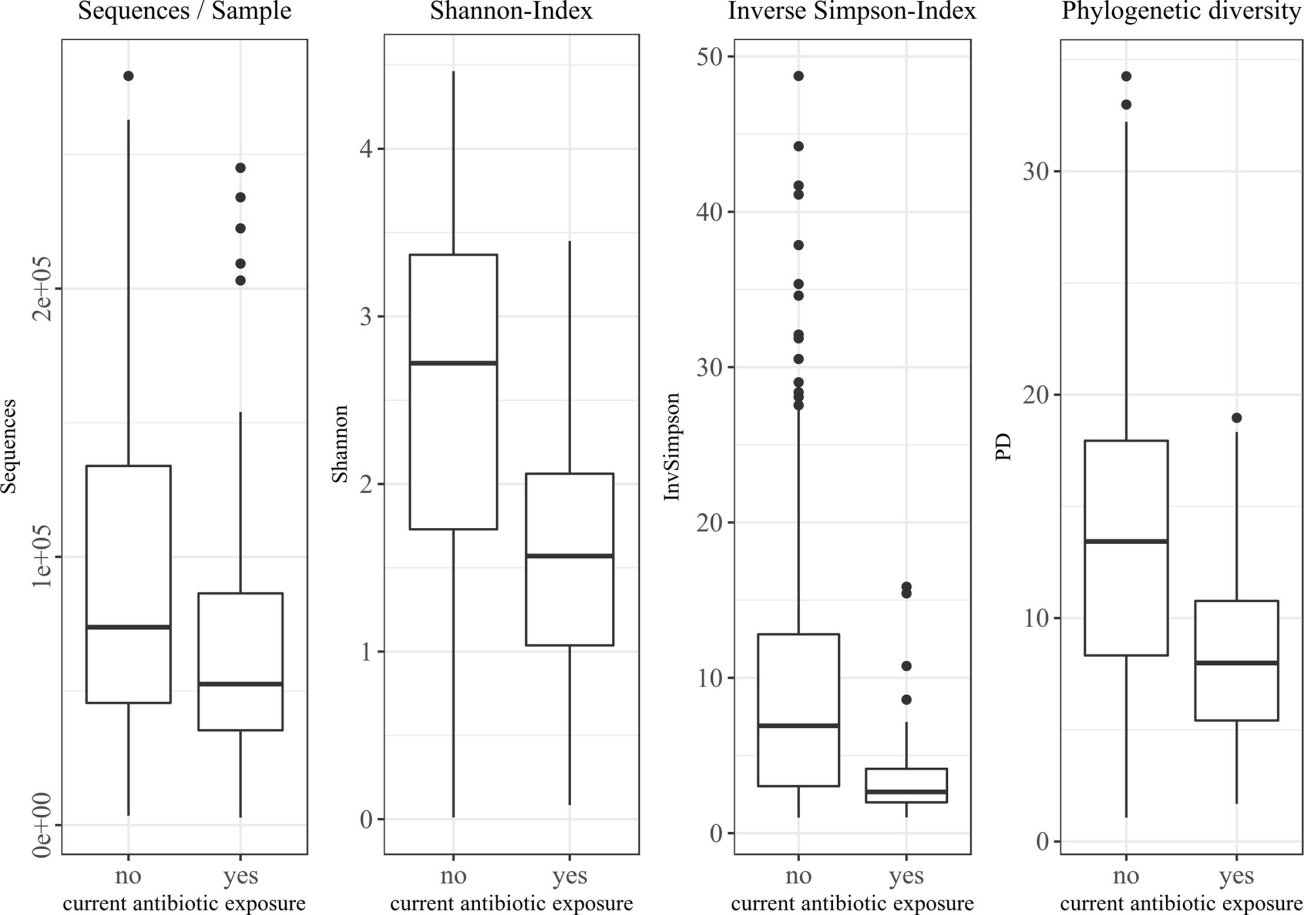
Effect of current antibiotic exposure on sequences per sample, Shannon-Index, inverse Simpson-Index and phylogenetic diversity (PD). No current antibiotic exposure: 310 samples; current antibiotic exposure: 95 samples.

The alpha diversity metrics of the analyzed swab samples are shown in Table 2. The Shannon index, the inverse Simpson index and the phylogenetic diversity of all samples were 2.28±1.11, 8.02±8.08 and 12.21±6,70, respectively. The Shannon index, as well as the phylogenetic diversity, were significantly increased in the baseline samples compared to samples obtained during the course of chemotherapy (*p* = 0.013 and *p*<0.001, respectively). Additionally, in samples from patients with current antibiotic exposure (n = 95) all three alpha diversity scores were significantly decreased (Table 3 and Fig 2).

### Bacterial domination in samples

Overall, bacterial domination, i.e. at least 30% of the sequences in a sample being assigned to a single taxon, was frequently observed both on family (338/405 samples; 83.5%) and genus level (231/405 samples; 57.0%). These dominations were caused by a variety of families and genera (Table 4), the most frequent one being Enterococcaceae (99 swabs, 24.4%; 31 patients, 75.6%) and *Enterococcus* (90 swabs, 22.2%; 28 patients, 68.3%), respectively. While the overall proportion of samples dominated by any genus or family did not differ between baseline and post-baseline samples, there was a significant shift towards domination by the genus *Enterococcus* in samples taken after day 0 (*p* = 0.01; Table 4).

**Table 4:** Most frequent dominating taxa on family and genus level.

	Dominating taxon	Percentage of samples (n); n = 405	Percentage of patients (n); n = 41
**Family level**	Enterococcaceae	24.4 (99)	75.6 (31)
	Lachnospiraceae	15.3 (62)	53.7 (22)
	Ruminococcaceae	8.1 (33)	46.3 (19)
	Prevotellaceae	7.4 (30)	36.6 (15))
	Enterobacteriaceae	6.7 (27)	43.9 (18)
	Other families^a^	21.5 (87)	75.6 (31)
	Total	83.5 (338)	100 (41)
**Genus level**	*Enterococcus*	22.2 (90)	68.3 (28)
	*Prevotella*	7.4 (30)	34.1 (14)
	*Corynebacterium*	4.2 (17)	29.3 (12)
	*Lactobacillus*	3.7 (15)	24.4 (10)
	*Bacteroides*	3.7 (15)	17.1 (7)
	Other genera^b^	15.8 (64)	63.4 (26)
	Total	57.0 (231)	95.1 (39)

^a^Other families included: Anaeroplasmataceae, Bacteroidaceae, Bifidobacteriaceae, Campylobacteraceae, Corynebacteriaceae, Dethiosulfovibrionaceae, Erysipelotrichaceae, Lactobacillaceae, Mycoplasmataceae, Porphyromonadaceae, Pseudomonadaceae, Staphylococcaceae, Veillonellaceae, Verrucomicrobiaceae

^b^Other genera included: *Akkermansia*, *Bifidobacterium*, *Blautia*, *Campylobacter*, *Dorea*, *Faecalibacterium*, *Fusobacterium*, *Mycoplasma*, *Porphyromonas*, *Pseudomonas*, *Pyramidobacter*, *Ruminococcus*, *Staphylococcus*, *Streptococcus*, *Ureaplasma*, *Veilonella*

### Detection of skin microbiota in rectal swab samples

An increased abundance of more than 10% of one of the genera *Corynebacterium*, *Staphylococcus* and *Streptococcus* was present in 99 samples (24.4%) from 34 different patients (82.9%). These genera are abundant taxa of the human skin microbiota [28]. Domination by one of these genera was present in 44 samples (10.9%) from 23 patients (56.1%). Of note, in half of the samples with skin bacterial dominations there was a shift towards the dominating genus seen in the previous or subsequent sample (Table 5).

**Table 5:** Increased relative abundance of and domination by common skin microbiota.

		Percentage of samples (n); n = 405	Percentage of patients (n); n = 41
*Corynebacterium*			
	Relative abundance in sample >10%	12.8 (52)	51.1 (21)
	Domination by this genus	4.2 (17)	29.3 (12)
	Shift towards domination seen in previous or subsequent sample^a^	3.7 (15)	26.8 (11)
*Staphylococcus*			
	Relative abundance in sample >10%	6.4 (26)	34.1 (14)
	Domination by this genus	3.5 (14)	14.6 (6)
	Shift towards domination seen in previous or subsequent sample^a^	1.7 (7)	2.4 (1)
*Streptococcus*			
	Relative abundance in sample >10%	10.4 (42)	58.5 (24)
	Domination by this genus	3.2 (13)	29.3 (12)
	Shift towards domination seen in previous or subsequent sample^a^	1.2 (5)	12.2 (5)
Any of the three genera			
	Relative abundance in sample >10%	24.4 (99)	82.9 (34)
	Domination by this genus	10.9 (44)	56.1 (23)
	Shift towards domination seen in previous or subsequent sample^a^	6.7 (27)	39.0 (16)

^a^Abundance of respective genus >10% in sample from same patient directly before or after sample with domination.

## Discussion

In this analysis, we present an intestinal microbiome analysis from a longitudinal cohort study utilizing deep rectal swabs for intestinal microbiota profiling. Previous studies have already compared rectal swabs with samples from different origins [6–9]. We validated the pipeline for DNA extraction and 16S rRNA gene sequencing on rectal swabs and stools obtained from seven healthy volunteers and one patient, confirming reproducibility of the results. Between swab- and stool-derived microbiota profiles we found some small differences, which may partly be due to dissimilarities in the sampled body sites (rectal mucosal wall versus stool), as previously reported [33]. Since it was not our aim to prove equality of these two sampling techniques, only this small number of comparative specimen from volunteers or patients were collected. Furthermore, the relatively low number of contaminant reads found in sterile swabs and negative controls is within the range of previously reported contamination of laboratory consumables [29], and is therefore expected not to confound the taxonomic composition of the patient samples.

Studies using stool samples are often confronted with low patient compliance and difficulties to obtain and process the required samples in a predefined time window. Hence, these studies usually face missing data as an issue. Over a period of 9 months, we were able to collect 418 of 431 (97%) intended samples as per collection protocol. Unfortunately, few studies have so far reported the actual adherence to their sample collection protocol, but, as an example, the exclusion of up to 16% of patients due to insufficient samples possibly indicates adherence problems [27]. From our experience, the sample yield in the present study is very high, demonstrating a practical advantage of rectal swabs over stool samples. There were no adverse events related to obtaining deep rectal swabs in this patient cohort. However, the current study was not designed to assess safety and we are not aware of other studies systematically assessing adverse events of this sampling technique in a similar patient cohort. Nevertheless, rectal swabs are broadly implemented in hematological and oncological departments in Germany for several years now, further supporting our safety observations.

A major concern associated with the use of rectal swabs relates to the recovered amount of bacterial gDNA for successful taxonomic profiling. The DNA quantity extracted from swab samples showed very divergent values including negative ones. However, we found no relation between the amount of DNA and sequencing output. This suggests that differences in DNA yields were caused by technical issues producing inaccurate negative values, i.e. the phenol and ethanol used during DNA isolation might have interfered with the signal intensity of the DNA quantitation assay, producing inaccurate negative values. After omitting samples with less than 2,000 reads, 405 of 418 samples (96.9%) could be included for further analyses. In comparison to previous studies performed in the setting of hematological and oncological patients, our rectal swab processing and sequencing yielded even a higher number of reads per sample than in stool samples [27, 34]. Importantly, the amount of DNA extracted from most of the rectal swabs (range 600 pg—5.5 μg), after depletion of host DNA, might allow metagenome sequencing (e.g. on an Illumina platform), which nowadays produces high quality data with as little as 10 pg of starting material [35, 36].

In terms of microbiota composition, our results can be compared with published studies, with the limitation that those were conducted using stool samples and analyzing different 16S rRNA variable regions. In a recent stool sample-based study comprising 34 patients receiving induction chemotherapy for the treatment of acute myelogenous leukemia [34], a wide range of alpha diversity among baseline samples was observed, with a mean Shannon index of 2.0 (95% confidence interval (CI), 1.7–2.4; range, 0.1–3.5). Similarly, in the present cohort, the mean Shannon index at baseline was 2.7 (95% CI, 2.4–3.0; range 0.01–4.5). The observed rates of domination on family (83.5% of samples) and genus level (57% of samples) are also comparable to their reported rate, with domination found in 79.2% of the samples [34]. In our cohort, Enterococcal domination was the most frequently observed one, as previously reported [27, 34]. In line with previous studies, we observed a statistically significant decrease in the microbial diversity over the course of chemotherapy [11, 27, 34] and after antibiotic exposure [12, 27, 34].

Our analysis, however, also revealed a possible difference in the microbiome composition from different intestinal locations captured by the use of a swab as compared to stool samples. We identified a relatively high number of patient samples in which typical skin commensals (*Corynebacterium*, *Staphylococcus*, *Streptococcus*) were present or even dominated the microbiota profile of the samples. This difference was not seen in the validation samples from a patient and various healthy volunteers. While we cannot rule out that these taxa represent actual skin microbiota of the patients resulting from flawed sampling technique, one could hypothesize that the high antibiotic burden and ongoing chemotherapy led to a shift towards these taxa in the distal intestinal microbiome. In half of the cases with observed domination by skin commensals, we found an increased abundance of the respective genus in the previous or subsequent sample, suggesting a true and possibly treatment-related change in the microbiota composition of the upper rectal mucosa. Interestingly, high relative abundances of skin bacteria, most frequently *Staphylococcus* spp. and *Streptococcus* spp., were also observed in studies using stool samples [27, 34]. *Corynebacterium* was reported to be more frequently found in rectal mucosal biopsies than stool samples in one study performing 16S rRNA gene sequencing on samples from healthy volunteers [33], suggesting that they may not only be part of the anal skin flora but also that of the rectal mucosa. Further studies are needed to address this issue including a larger number of comparative stool and rectal swab samples from patients.

Our study shows that the utilization of rectal swabs is adequate and feasible for the analysis of intestinal microbiota compositions in patients with exposure to chemotherapy and antibiotics. The characteristics of the rectal swab sample collection, including diversity metrics and domination frequencies, are comparable with those of similar clinical cohorts using stool samples. Our results confirm previous findings about the impact of chemotherapy and antibiotic exposure on microbial diversity. The increased abundance of skin commensals may possibly be related to chemotherapy and antibiotic exposure in this specific cohort, rather than contamination with skin bacteria during sampling. This observation needs to be investigated in future studies in more detail. In conclusion, rectal swabs have a number of practical advantages possibly leading to higher sample adherence and patient compliance. The total amount of DNA recovered from rectal swabs should also be sufficient for metagenome sequencing, which could provide more comprehensive insights into the functional and metabolic potential of the microbial community.

## Supporting information

S1 Fig Reproducibility and consistency of microbiota profiles. (A): Taxonomic profiles and respective legend at the family level of different technical replicates from swabs and stools of volunteer H1. (B): Beta-diversity calculated as weighted Unifrac and visualized by principal coordinate analysis (PCoA).
(PDF)

S2 Fig Impact of Cary-Blair medium on microbiota profiles. (A): Taxonomic profiles and respective legend at the family level of mock and volunteer samples transported in both Cary-Blair medium and saline solution (NaCl). (B): Beta-diversity calculated as weighted Unifrac and visualized by principal coordinate analysis (PCoA). The mock community is composed of 12 bacterial species belonging to 10 families.
(PDF)

S1 File List of commands used for bioinformatics processing of raw data and microbiome analyses. (TXT)

S2 File Fasta control sequences. (FNA)

S1 Table Characteristics of the samples used for pipeline validation. (XLSX)

S2 Table Dual-indexed PCR amplification primer sequences. Red: Illumina adapter sequence. Black: variability region. Green: specific sample barcode. Blue: forward primer targeting the specific 16S rRNA regions V3-V4. Purple: reverse primer targeting the specific 16S rRNA region V3-V4.
(XLSX)

S3 Table Information and taxonomy classification of the control samples. (XLSX)

S4 Table List of sequenced swab samples and relative information. (XLSX)
