# Supplementary figures and images for: Usability of rectal swabs for microbiome sampling in a cohort study of hematological and oncological patients
Source: PLoS One. 2019 Apr 15;14(4):e0215428. doi: 10.1371/journal.pone.0215428 (PMC6464231; doi:10.1371/journal.pone.0215428)

S1 Fig: Reproducibility and consistency of microbiota profiles

A

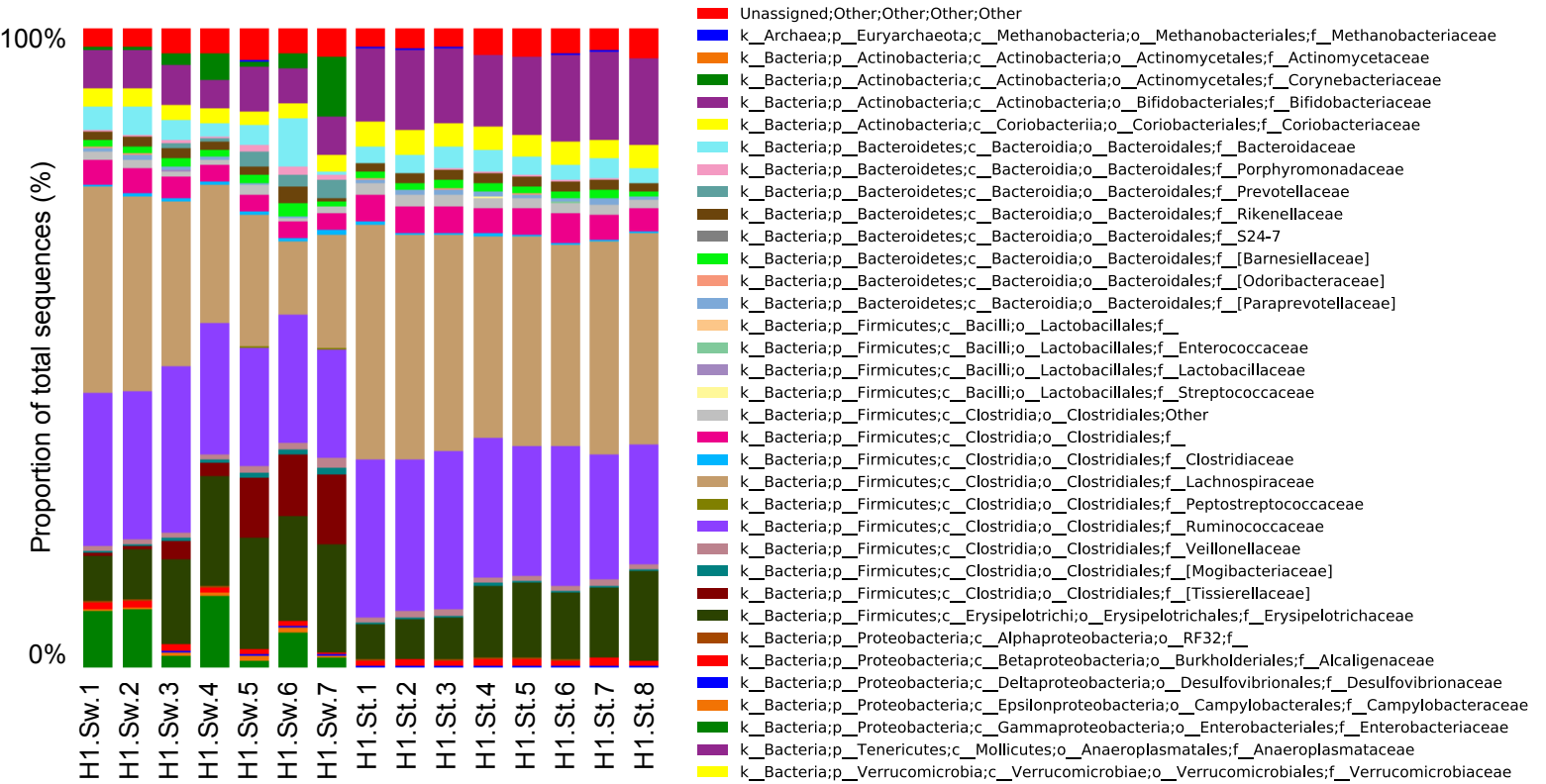

B

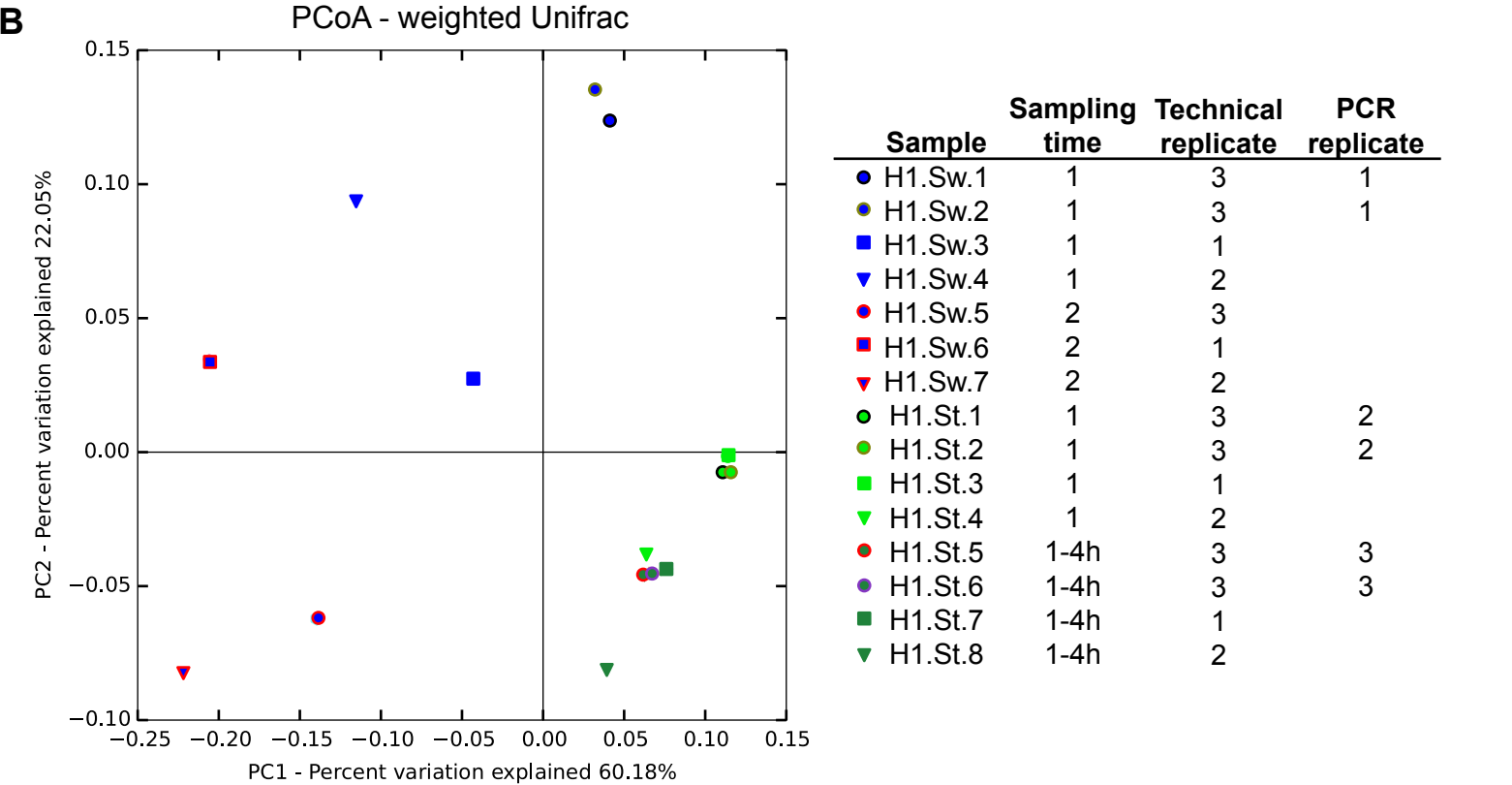

Supplement: S1 Fig — (A): Taxonomic profiles and respective legend at the family level of different technical replicates from swabs and stools of volunteer H1. (B): Beta-diversity calculated as weighted Unifrac and visualized by principal coordinate analysis (PCoA). (PDF) [file pone.0215428.s001.pdf]

S2 Fig: Impact of Cary-Blair medium on microbiota profiles

A

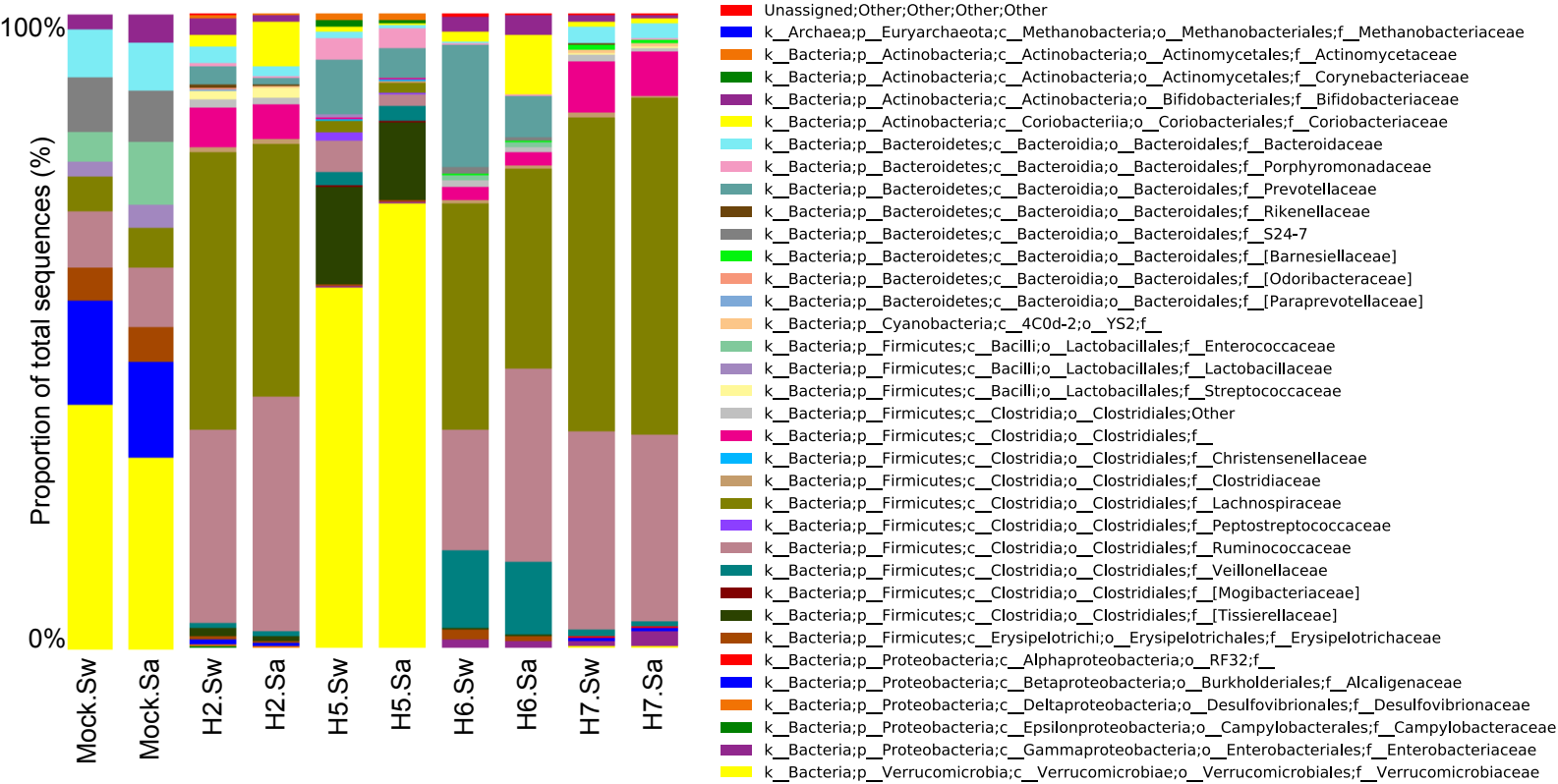

B

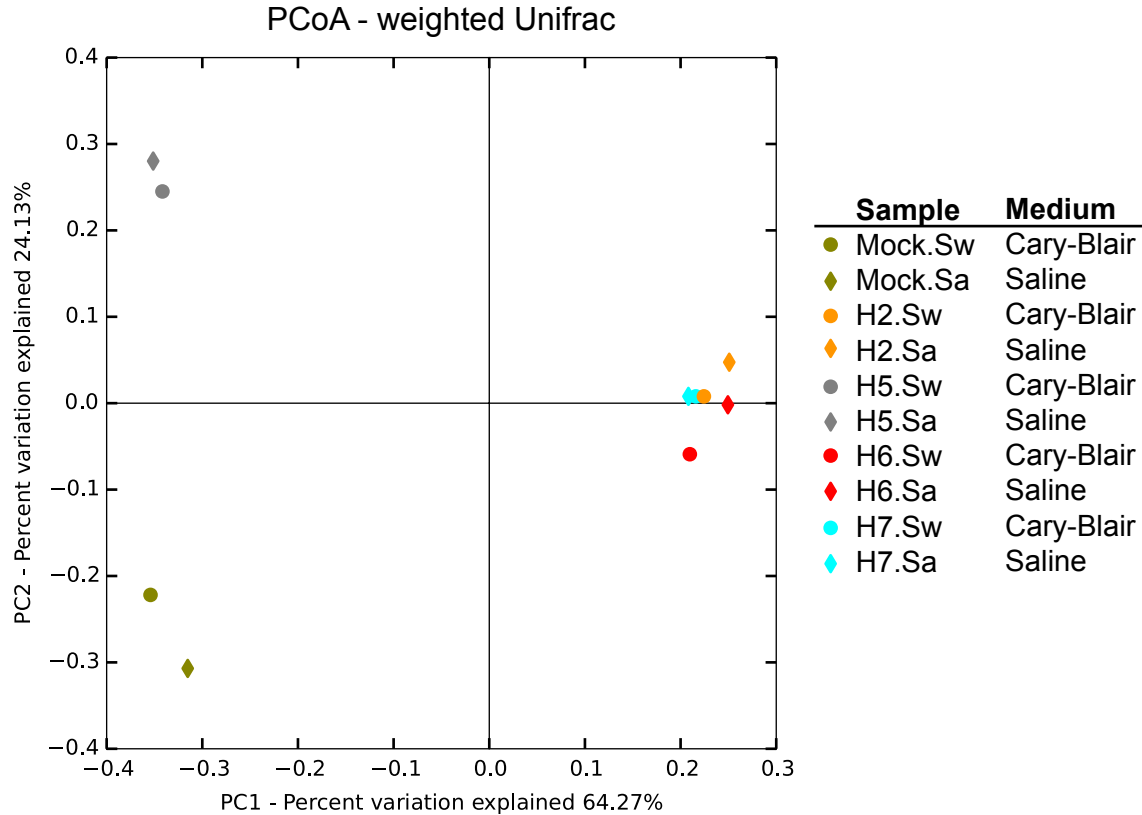

Supplement: S2 Fig — (A): Taxonomic profiles and respective legend at the family level of mock and volunteer samples transported in both Cary-Blair medium and saline solution (NaCl). (B): Beta-diversity calculated as weighted Unifrac and visualized by principal coordinate analysis (PCoA). The mock community is composed of 12 bacterial species belonging to 10 families. (PDF) [file pone.0215428.s002.pdf]
